# Effect of application of short-form video health education on the health knowledge and satisfaction with nursing care of patients with lower extremity fractures

**DOI:** 10.1186/s12912-023-01530-3

**Published:** 2023-10-20

**Authors:** Yuliu Zheng, Qiuyan Yan

**Affiliations:** 1 The Nursing Department of the First Affiliated Hospital of Fujian Medical University, 20 Cha Zhong Lu, Taijiang District, RN 350004 Fuzhou, China; 2 Fujian Medical University School of Nursing, Fuzhou, 350004 China

**Keywords:** Lower extremity fracture, Short video, Health education, Perioperative nursing care

## Abstract

**Background:**

Video health education has been increasingly adopted in the clinic to expand patient guidance and improve perioperative outcomes. To date, however, it is unclear whether the use of short-form videos and web-based clinician-created forums actually improve the perioperative experience of patients.

**Objective:**

To evaluate the effect of application of short-form video health education on the health knowledge and satisfaction with nursing care of patients with lower extremity fractures.

**Methods:**

This study is a quasi experimental study, using convenient sampling method and grouping according to historical control., one hundred and twenty-two patients admitted with lower limb fractures to the orthopedic ward of a tertiary first-class general hospital in Fujian, China were enrolled in this study. Based on their admission order, patients admitted from September 2021 to November 2021 were enrolled into the control group (*n* = 61) and patients admitted from December 2021 to March 2022 were enrolled in the intervention group (*n* = 61). Both groups received identical admission-based patient education, perioperative care, and discharge rehabilitation instructions. The control group received the traditional education method while the intervention group received a short-form educational video in addition to the traditional education method. Patient perioperative health knowledge and patient satisfaction with nursing care during treatment of lower limb fractures were compared across the two groups.

**Results:**

Preoperative health knowledge in the intervention group was 95.1%, compared to 82.0% in the control group (*χ*^2^ = 3.954, *P*<0.05). The Newcastle satisfaction with nursing scales score in the intervention group was (80.902 ± 7.016) points, compared to (78.131 ± 8.213) points in the control group. These group differences were statistically significant (*P* < 0.05).

**Conclusion:**

The application of a short-form educational video during the perioperative care of patients with lower limb fractures may improve patients’ understanding of perioperative health and increase satisfaction with nursing care.

## Introduction

Patients with lower extremity fractures often suffer from acute injuries with severe pain. Subsequently, the self-care ability and quality of life of the patients are reduced [1]. Surgery is the main treatment modality for lower extremity fractures. As such, patients require postoperative bed rest and increased hospital stays, leading to high incidences of complications such as non-suppurative inflammation of lower extremity veins. Reasonable guidance and functional exercise should be provided depending on the patient’s condition. An effective nursing program can improve the quality of life of patients during the perioperative period, improve their negative emotions, and promote the recovery process [2]. The method of routine oral education has certain limitations, including but not limited to the scope and expression of the content, the duration of the education, the patient’s memory and the ability to understand the health education, all of which have an impact on the effect of the health education [3]. Govender et al. applied video education to patients undergoing head and neck cancer radiation therapy, and the results showed that it can effectively improve the accuracy of oral function exercises and reduce the incidence of complications associated with head and neck cancer radiation therapy [4]. Denny MC used short-video education in stroke caregivers, and the video education method increased the enthusiasm of patients and caregivers to receive health education, ensured the accuracy of the transmission of health education content, and improved the caregivers’ caregiving ability. More studies have shown that the application of short-video format health education during the course of perioperative care can improve the patients’ understanding of their condition and increase enthusiasm for exercise and patients’ satisfaction with their nursing care [5–7]. Compared to other forms of health education, video education offers intuitive guidance, a wide range of applications, and is easily accessible for widespread use. It represents a novel auxiliary health education method that utilizes videos as an information medium, making it easy for patients with lower limb fractures to comprehend and efficiently receive relevant information regarding perioperative care and rehabilitation exercises. It is a valuable area for research and promotion. In order to explore the impact of a short-form health education video in perioperative patients with lower extremity fractures, this study aimed to explore the effects of this teaching tool through patient-centered outcomes.

## Methods

### The Study Design and participants

Convenience sampling was used to select 122 patients with lower limb fractures admitted to a Grade A hospital in Fujian province from September 2021 to March 2022. A historical control group (61 cases) comprised patients admitted from September to November 2021, while an intervention group (61 cases) included patients admitted from December 2021 to March 2022. Inclusion criteria: ages 18–75, CT-diagnosed lower limb fractures requiring surgery, WeChat users, and voluntary participation. Exclusion criteria: concomitant cranio-cerebral injury, impaired consciousness, mental disorders, lower limb venous inflammation, inability to cooperate, or early withdrawal from the study.

### Sample Size and Data Collection

For robust statistical power, we calculated the sample size using Gpower 3.1 software. With d = 0.5 (moderate effect size), 1-β = 0.80 (statistical power), and α = 0.05 (significance level), a minimum of 128 participants (64 in each group, 1:1 ratio) was required. We distributed 128 questionnaires, collected 122 (95% response rate), and excluded six participants lost to follow-up. Self-administered questionnaires gathered demographic and medical data. All data were collected anonymously and securely to ensure confidentiality.

### Group Assignment

The control group (61 cases) received traditional patient education, while the intervention group (61 cases) received a concise patient education video. Control group patients were admitted from September to November 2021, and intervention group patients were admitted from December 2021 to March 2022.

### Intervention methods

In the control group, traditional patient education involved oral and paper-based instruction conducted at admission, the day before surgery, the day of surgery, the day after surgery, and the day before discharge. It included addressing patient queries and demonstrating exercises. Questionnaires were filled out one day before discharge, based on electronic health records and provider confirmation.In the intervention group, patients watched department-produced health videos alongside their regular education. The specific process was as follows: (1) The head nurse organized the production of a concise health education video. A nursing forum was created on the department’s official WeChat account, where patient education content was customized based on patient characteristics, medical staff experience, and existing literature (2). The nursing team created concise 1–3 min videos filmed in familiar hospital locations, enhancing clinical understanding. The videos covered perioperative care for lower extremity fracture patients, including admission instructions, ward setup, appointments, billing, specimen collection, sputum management, blood drawing precautions, pre and postoperative care, limb exercises, pain management, instrument usage, and discharge guidance, totaling 14 videos (3). Implementation method: Nurses were trained to guide patients and families in using WeChat. A registered nurse scheduled patient log-ins at admission, before surgery, post-surgery, and pre-discharge [8]. Attending nurses addressed questions, enhanced education quality, and promoted independent learning. An on-site questionnaire was completed before patient discharge, verified by electronic health records and discharge time (4).

In the feedback phase, registered nurses collected input from patients and families to enhance the short-form video health education and nursing care experience.

### Data collection and tools

Perioperative health education knowledge was assessed using the “Health Education Knowledge Awareness Questionnaire,“ which included 9 items covering various aspects of health education. Scores ranged from 1 (poor) to 5 (excellent), with a total score of ≥ 32 points indicating acceptable knowledge. Cronbach’s alpha coefficient for the experience scale was 0.943 in this study.Newcastle Satisfaction with Nursing Scale (NSNS) developed by THOMAS [8] and others [9], measures patient satisfaction with nursing services during hospital stays using 19 items scored on a 5-level Likert scale. Higher scores reflect increased satisfaction with perioperative nursing care. The Cronbach’s alpha coefficient for experience scale was 0.967, and the correlation between items was 0.53–0.82. In this study, the Cronbach’s alpha coefficient for experience scale was measured to be 0.932.

### Quality control

We implemented quality control measures to maintain data accuracy, objectivity, and authenticity. Personnel underwent standardized training, ensuring proficiency in procedures and experimental processes. Surveys were conducted in quiet environments, and assistance was provided for those with reading difficulties. Completed questionnaires were carefully reviewed for accuracy before data analysis.2.7Statistical methods.

SPSS 26.0 was used to evaluate the data, and descriptive statistics were used to analyze the demographic characteristics of the research objects. Continuous variables were expressed as percentages, and the χ2 test was used to compare values between groups. Data conforming to normal distribution were expressed as mean ± standard deviation (x ± s), and two independent samples t -tests were used to compare the means between two groups. *P* < 0.05 was regarded as a statistically significant difference.

## Results

### Comparison of demographic characteristics between the two groups

There was no significant difference between the two groups in age, gender, medical payment method, educational level, fracture site, etc. (*P* > 0.05). For details, see Table 1.

**Table 1:** Comparison of demographic characteristics of patients with lower extremity fractures between two groups [cases (percentage, %)]

item	control(n = 61)	intervention(n = 61)	*χ* ^2^	*P*
Age (years)			0.32	0.451
18–55	24(39.3%)	20(32.8%)		
56–75	37(60.7%)	41(67.2%)		
Sex			0.525	0.365
Male	29(47.5%)	34(55.7%)		
Female	32(52.5%)	27(44.3%)		
Medical payment method			0.764	0.682
Medical insurance	38(62.3%)	42(68.9%)		
At own expense	21(34.4%)	18(29.5%)		
Commercial insurance	2(3.3%)	1(1.6%)		
Educational level			5.04	0.169
Primary school	19(31.1%)	25(41.0%)		
Middle school	33(54.1%)	21(34.4%)		
High school	2(3.3%)	4(6.6%)		
College and above	7(11.5%)	11(18.0%)		
Fracture site			6.849	0.335
Hip	19(31.1%)	22(36.1%)		
Pelvis	2(3.3%)	4(6.6%)		
Femoral shaft	4(6.6%)	6(9.8%)		
Tibia and fibula	3(4.9%)	8(13.1%)		
Patella	17(27.9%)	13(21.3%)		
Calcaneus	5(8.2%)	2(3.3%)		
Ankle	11(18.0%)	6(9.8%)		

### Comparison of health education knowledge between groups of patients with lower extremity fractures

The health education knowledge score in the intervention group was 95.1%, higher than 82.0% in the control group (*P* < 0.05). There were significant differences between the two groups across five items specifically: hospital environment and precautions, precautions before and after examination or operation, rehabilitation knowledge of disease, methods of promoting rehabilitation, and overall health education (*P* < 0.05). There were no significant differences (P > 0.05) between the two groups in terms of treatment and nursing precautions, medication guidance, dietary guidance, and post-discharge rehabilitation precautions. See Tables 2 and 3 for details.

**Table 2:** Health education knowledge and score comparison between groups of lower extremity fracture patients (‾x ± s). The asterisk “*” indicates the degree of significance of the test. Generally speaking, one star represents significance at the 0.05 level, indicating 90% significance; two stars represent significance at the 0.01 level, indicating 95% significance; and three stars represent significance at the 0.001 level, indicating 99% significance

Group	Awareness score	Awareness rate
control(n = 61)	36.213 ± 4.446	50(82.0%)
intervention(n = 61)	38.311 ± 4.353	58(95.1%)
*χ²/t*	-2.634^1)^	3.954^2)^
*P*	0.010^***^	0.023^**^

note: 1)t value; 2) χ^2^ value

**Table 3:** Comparison of scores of perioperative health knowledge between groups of patients with lower extremity fractures (‾x ± s). The asterisk “*” indicates the degree of significance of the test. Generally speaking, one star represents significance at the 0.05 level, indicating 90% significance; two stars represent significance at the 0.01 level, indicating 95% significance; and three stars represent significance at the 0.001 level, indicating 99% significance

item	control(n = 61)	intervention(n = 61)	*t*	*P*
Hospital environment and precautions	3.967 ± 0.632	4.262 ± 0.545	-2.763	0.007^***^
Precautions before and after examination or surgery	3.918 ± 0.614	4.230 ± 0.589	-2.861	0.005^***^
Treatment and Nursing Precautions	4.033 ± 0.632	4.164 ± 0.583	-1.192	0.236
Disease Rehabilitation Knowledge	3.787 ± 0.635	4.180 ± 0.563	-3.62	0.000^***^
Medication guidance	4.180 ± 0.592	4.197 ± 0.628	-0.148	0.882
Guidance on ways to promote recovery from illness	3.918 ± 0.526	4.361 ± 0.578	-4.423	0.000^***^
dietary guidance	4.443 ± 0.646	4.508 ± 0.566	-0.596	0.552
Rehabilitation and discharge precautions	4.131 ± 0.695	4.328 ± 0.625	-1.644	0.103
General situation of health education	3.836 ± 0.522	4.082 ± 0.458	-2.765	0.007^***^

### Comparison of newcastle satisfaction with nursing scale scores between groups of patients with lower extremity fractures

The satisfaction score of the intervention group (80.902 ± 7.016) was higher than that of the control group (78.131 ± 8.213), and the difference was statistically significant (*P* < 0.05). There were significant differences between the two groups across six items specifically: time nurses spent on patient, nurses’ work ability, the amount of disease and treatment information provided, nurse put relatives’ mind at rest, the type of disease and treatment information provided, and the nurses’ awareness of patients’ needs (*P* < 0.05). There were no significant differences (P > 0.05) between the two groups across thirteen items, including nurse’s availability, the nurse’s knowledge about the patient’s care, how quickly nurses responded to patient, nurses’ treatment of you as an individual, how often nurses checked on a patient, nurse helpfulness, how nurses explained things to patient, nurses’ manner while doing their work, respectful attitude towards patients in the nursing process, nurses listened to patients’ worries, freedom nurses gave patient in the ward, the willingness to respond to patient requests, and the level of privacy nurses gave patient. See Tables 4 and 5 for details.

**Table 4:** Comparison of Newcastle Satisfaction with Nursing Scale scores between two groups of patients with lower extremity fractures (‾x ± s). The asterisk “*” indicates the degree of significance of the test. Generally speaking, one star represents significance at the 0.05 level, indicating 90% significance; two stars represent significance at the 0.01 level, indicating 95% significance; and three stars represent significance at the 0.001 level, indicating 99% significance

Group	NSNS score
control(n = 61)	78.131 ± 8.213
intervention(n = 61)	80.902 ± 7.016
*t*	-2.003
*P*	0.047^**^

**Table 5:** Comparison of scores on each item of the Newcastle Satisfaction with Nursing Scale between two groups of patients with lower extremity fractures (‾x ± s). The asterisk “*” indicates the degree of significance of the test. Generally speaking, one star represents significance at the 0.05 level, indicating 90% significance; two stars represent significance at the 0.01 level, indicating 95% significance; and three stars represent significance at the 0.001 level, indicating 99% significance

item	Control(n = 61)	Intervention (n = 61)	*t*	*p*
Time nurses spent with patient	3.984 ± 0.671	4.328 ± 0.676	-2.823	0.006^***^
How capable nurses were at their job	4.131 ± 0.645	4.443 ± 0.646	-2.665	0.009^***^
Nurses are always available if needed	4.000 ± 0.606	4.033 ± 0.515	-0.322	0.748
Nurse’ knowledge about patient care	4.180 ± 0.563	4.230 ± 0.529	-0.497	0.620
How quickly nurses responded to patient	3.869 ± 0.695	4.016 ± 0.591	-1.263	0.209
Nurses’ treatment of you as an individual	3.967 ± 0.632	4.082 ± 0.640	-0.997	0.321
Amount of information nurses gave patient	4.098 ± 0.651	4.393 ± 0.613	-2.578	0.011^**^
How often nurses checked on patient	4.311 ± 0.564	4.311 ± 0.534	/	1
Nurse helpfulness to patient	4.213 ± 0.661	4.295 ± 0.587	-0.724	0.471
How nurses explained things to patient	4.180 ± 0.671	4.344 ± 0.602	-1.42	0.158
Nurses put relatives’ mind at rest	4.246 ± 0.596	4.475 ± 0.566	-2.181	0.031^**^
Nurses’ manner while doing their work	4.377 ± 0.610	4.508 ± 0.566	-1.231	0.221
Type of information nurses gave patient	4.148 ± 0.543	4.492 ± 0.566	-3.428	0.001^***^
Respectful attitude towards patients in the nursing process	4.328 ± 0.598	4.393 ± 0.525	-0.644	0.521
Nurses listened to patients’ worries	4.115 ± 0.580	4.180 ± 0.619	-0.603	0.547
Freedom nurses gave patient in the ward	3.672 ± 0.473	3.623 ± 0.489	0.565	0.573
Nurses’ willingness to respond to patients’ needs	4.016 ± 0.671	4.197 ± 0.601	-1.565	0.120
Privacy nurses gave patient	4.262 ± 0.630	4.279 ± 0.581	-0.149	0.881
Nurses’ awareness of patient needs	4.033 ± 0.576	4.279 ± 0.581	-2.346	0.021^**^

## Discussion

### Short-form health education video implementation improves the perioperative health knowledge of patients with lower extremity fractures

The results of this study demonstrated that short-form health education videos were beneficial toward the improvement of perioperative health knowledge in patients with lower extremity injuries. This was particularly attributed to five aspects, including hospital environment and precautions, precautions before and after inspection or operation, knowledge of disease rehabilitation, methods of promoting disease rehabilitation, and overall health education. Comparison between the control and intervention groups revealed that the scores of the intervention group were significantly higher than those of the control group. These were particularly attributed to the section “hospital environment and precautions”. One of the reasons for this difference may be that most of the patients enrolled suffered from great pain due to the fractures caused by external factors when they were first admitted to the hospital. It is plausible that the pain focus on admission perturbed their ability to concentrate on the nurses’ admission education. Patients often required bed rest after admission and were unfamiliar with the surroundings environment, due to the inability to get out of bed and move around. However, the patients in the intervention group had access to the short-form videos, allowing them to learn about the department environment and other precautions despite hinderances to receiving conventional education on admission. This hypothesis is consistent with the findings of other reports [10, 11], who suggested that patients’ awareness of the ward environment was greatly improved after receiving video education. The item “precautions before and after examination or operation” may operate under a similar phenomenon, whereby routine, oral education may be incompletely absorbed due to patient anxiety, which tends to be higher when the patient is unfamiliar with the process. Indeed, patients repeatedly asked the nurses about the precautions and the experiences of the people around them. Similarly, postoperative patients are often distracted due to postoperative pain, which can limit their ability to absorb oral education. By providing short-form video health education, patients can intuitively understand the procedure ahead of the surgery, reducing perioperative anxiety and circumventing the absorption of pertinent information at inopportune times. This hypothesis is similar to some previous reports [12–14]. Oliveira AP similarly found that the application of preoperative video resources can more effectively preserve the patients’ retention of the content, supporting the findings of this study [15]. Regarding the item “disease rehabilitation knowledge”, conventional education methods are generally conducted by medical staff to guide patients in rehabilitation exercise at a specified time point. By the end of the education, patients often forget steps and require guided re-training, especially for lengthy rehabilitation processes. In this study, the intervention group was able to review the processes autonomously via the short-form videos, accessing a preview of the rehabilitative process and reviewing the experience repeatedly if needed. This is consistent with the point of view which evaluated the application of video education in patients after orthopedic surgery. For the item “methods of promoting disease recovery”, it is plausible that compared with the conventional educational method, the novelty of short-form video health education increases interest and engagement. This hypothesis is consistent with the research results, wherein three-dimensional and multi-form nursing and health education increased patient buy-in. In this study, there was no significant difference between groups on the items “treatment and nursing precautions”, “medication guidance”, “dietary guidance”, or “rehabilitation and discharge precautions”. The reasons for this may be that these basic nursing measures are routine in the daily care of patients, and nurses implement this conventional oral education with such regularity that presentation of this education in short-form video may not be perceived as any different by the patient. Due to the lack of pre- and post-intervention comparisons in this study, the data was not able to be evaluated according to change in baseline health knowledge between the control group and the observation group using different health education methods. As such, the research design can be further improved in the future.

### Short-form video health education improves the satisfaction of patients with lower extremity fractures during hospitalization

As can be seen from Tables 4 and 5, the Newcastle satisfaction with nursing score of the intervention group was higher than that of the control group. This was specifically reflected in the degree of satisfaction with the time spent by nurses, how capable nurses were at their job, the amount of disease and treatment information provided, nurses ability to put relatives’ mind at rest, the type of disease and treatment information provided, and nurses’ awareness of patient needs. Patients with lower extremity fractures tend to be bedridden for long periods of time. Capitalizing on this scenario, short-form health education videos can be leveraged to assist patients and their families with meaningful, semi-autonomous learning opportunities with patient-centered control of time and frequency access. This may help reduce patients’ anxiety and minimize obstruction to learning due to distractions (e.g. pain, anxiety, etc.). Additionally, familiarity with the clinical environment via frequent access to digital health education can circumvent anxiety related to unfamiliar environments, a chronic limitation of lower extremity patients with limited mobility. Finally, addition of short form health education videos and access to a nursing-led social media forum increase the patients’ perception of the nurses’ work ability and recognition of medical staff. This is similar to the findings of Wang’s and others who have evaluated the application of video health education [16, 17]. In the control group, traditional oral and paper-based materials for health education may be perceived as being of “lower effort” by the patients, which may translate into their perception of their clinical care experience in general. One limitation of this methodology is that a large fraction of patients with lower extremity fractures are elderly, and thus less likely to engage with digital forms of health education. However, these same patients are more likely to forget the content of conventional oral education, experience high anxiety/feel overwhelmed, and report lower satisfaction with nursing care. The results of this study indicate that efforts to diversify patient health education have promising outcomes, reiterating the need to champion conventional and novel methods for wide patient audiences of all ages. These findings are supported by previous research studies demonstrating improved patient satisfaction in light of video-centric patient health education [18, 19].

### Limitations of short form-video health education in orthopedic nursing work

During the implementation process, it was found that different patients have different needs. Some patients want more in-depth knowledge or have specific questions, while the production of videos mainly focuses on common problems and exercise methods. Additionally, patients’ acceptance of new types of education methods varies greatly. Using short form-videos for health education in the digital age requires continuous exploration of more suitable methods for patients.

### Advantages and limitations of this study

This study is a quasi-experimental study, which belongs to a prospective study where intervention precedes effect, and the causal relationship is highly credible with feasibility and practicality. It is commonly used in clinical nursing research, but lacks randomized grouping or a control group, making it difficult to fully attribute the effects to the intervention, resulting in lower credibility compared to experimental studies. In this study, the baseline surveys for the control group and intervention group were consistent. The surgical doctors and nursing staff in the department all met the same standards, ensuring stability and reducing bias due to the lack of random controls. At the same time, considering the comparison between the two groups during the same period, providing video education to the intervention group only may cause interference and produce a halo effect due to video sharing. Additionally, it may affect the mental health of the control group and their family members, causing psychological imbalance, etc. To circumvent these, the two groups were not investigated in parallel.

## Conclusion

In summary, short-form health education videos demonstrated a significant improvement in perioperative health knowledge and satisfaction with perioperative care in patients with lower extremity fractures. This study also demonstrated the feasibility of promoting health education videos to a widespread clinical audience, though it should be noted that this methodology may be of limited utility for elderly audiences, for whom compliance with technical aids may be a challenge.

## Data Availability

The data used to support the findings of this study are available from the corresponding author upon request.
